# Evaluation of the impact of COVID-19 in people coinfected with HIV and/or tuberculosis in low-income countries: study protocol for mixed methods research in Burkina Faso

**DOI:** 10.1186/s12879-023-08076-4

**Published:** 2023-02-22

**Authors:** Cheick Ahmed Ouattara, Armel G Poda, Ziemlé Clément Méda, Yacouba Sawadogo, Odilon Kabore, Emile Birba, Adama Sourabié, Jacques Zoungrana, Isidore Tiandiogo Traore, Ibrahim Sangaré, Awa Ymba, Sylvain Godreuil, Abdoul-Salam Ouedraogo

**Affiliations:** 1 Laboratoire des Pathogènes Émergents et Re émergents, Université Nazi BONI, Bobo- Dioulasso, Burkina Faso; 2 Centre Hospitalier Universitaire Souro SANOU, Bobo-dioulasso, Burkina Faso; 3 Centre Muraz, Bobo-Dioulasso, Burkina Faso; 4 Université Montpellier 1, Montpellier, France; 5 Centre Hospitalier Régional Universitaire de Montpellier, Montpellier, France; 6 Institut Supérieur des Sciences de la Santé, Université Nazi Boni, Bobo-Dioulasso, Burkina Faso

**Keywords:** COVID-9, HIV, Tuberculosis, Coinfection, Risk populations, Research protocol

## Abstract

**Background:**

An issue of particular concern is the impact of the 2019 novel coronavirus (2019 nCOV) on the people coinfected with the Human Immuno-deficiency Virus (HIV) and/or tuberculosis (TB). Unfortunately, this interaction has not been well explored in African despite the large proportion of these risk populations living with HIV and/or patients and/or tuberculosis (TB) in the African region. This study aims to design a research protocol for assessment of the impact of coronavirus disease 2019 (COVID-19) on these risk populations in response to COVID-19 strategic plans in Burkina Faso by generating serological, epidemiological, virological, clinical and socio-anthropological evidence-based data.

**Methods:**

A multidisciplinary research will be conducted in the city of Bobo-Dioulasso, Burkina Faso using mixed methods. Data will be collected from a cohort of people living with HIV and/or TB patients in the city (i) to determine the proportion of people with specific antibodies against the severe acute respiratory syndrome coronavirus 2 (SARS-CoV-2) using retrospective data ; (ii) to determine the proportion of people infected with Covid-19 and the dynamics of viral loads and antibodies in these people based on prospective data; (iii) to identify circulating SARS-COV-2 variants and novel biomarkers using prospective data ; (iv) to analyze perceptions, community experiences and response strategies during the public health emergencies imposed by COVID-19 through a qualitative study.

**Discussion:**

This study will generate factual and comprehensive data that will contribute in improving response strategies to COVID-19 and the other possible emerging diseases with keen interest on the risk populations living with HIV and/or TB infected patients.

**Supplementary Information:**

The online version contains supplementary material available at 10.1186/s12879-023-08076-4.

## Introduction

In December 2019, Severe Acute Respiratory Syndrome Coronavirus 2 (SARS-CoV-2) infection emerged in China and quickly spread worldwide to become one of the world greatest health challenges. With over eighteen million people infected and over five million deaths, the coronavirus disease 2019 (COVID-19) pandemic has revealed the extreme fragility of the world health systems in protecting the most vulnerable people such as the elderly population with long stay in healthcare facilities and suffering from chronic diseases [1–3]. The response to epidemic, particularly in low-income countries (LCI) has already been centered on limited diagnostic, management and research resources in the general population. This can lead to increased risks of morbidity and mortality in vulnerable populations and to a lack of evidence-based data on disease interactions. People living with the human immunodeficiency virus (PLWHIV) and/or TB patients (TBP) are also subjected to chronic [4–6] and they have been at the center of several scientific questions with the advent of COVID-19 pandemic. Indeed, modelling studies suggested that the COVID-19 pandemic could lead to an excess of 6 million TB-related and 0.5 million HIV-related deaths by 2025 due to the collapse of healthcare systems or the disruption of prevention and treatment services [7–9]. Available data on the possible increased risk of SARS-CoV-2 infection with severe forms in PLWHIV compared to the general population is evolving and [10]. Some studies reported that the susceptibility of PLWHIV to SARS-CoV-2 infection is comparable to that of the general population, even if the HIV infection is controlled (differentiation cluster 4 (CD4) count > 200 cells/mm^3^ and suppressed viral load (VL) whereas other student reported increased susceptibility of people living with HIV [11–13], [14]. Africa accounts for more than 70% (25.4 million) of [15] and more than 20% of new TB cases [8]. Although several mathematical models have estimated the possible impact of the SARS-COV-19 pandemic on patients with HIV, TB and [16–18], few studies have assessed the actual prevalence of the SARS-COV-2 infection in risk populations such as people living with the human immunodeficiency virus (PLWHIV) and/or tuberculosis (TB). Burkina Faso has been facing COVID-19 pandemic since March 2020 and the response to the pandemic is organized around a strategic control plan consisting mainly of the management of people suspected of SARS-CoV-2 or confirmed to be infected with SARS-CoV-2 in order to limit the spread of the disease. Recently, vaccination has also been included in the strategic control plan. Several research studies on COVID-19 are ongoing in Burkina Faso, ranging from clinical trials to sero-epidemiological studies and emergence of new SARS-CoV-2 variants [19]. In other countries around the world, several studies mainly targeted the general population to evaluate the impact of COVID-19 pandemic. This study aims to design a research protocol for assessment of the impact of coronavirus disease 2019 (COVID-19) on these risk populations in response to COVID-19 strategic plans in Burkina Faso by generating serological, epidemiological, virological, clinical and socio-anthropological evidence-based data.

The specific objectives of the study include:

- To determine the seroprevalence of SARS-CoV-2 and the viral load and antibody dynamics of COVID-19 in cohorts of people living with HIV (PLWHIV) and/or TBP.
- To identify specific biomarkers of SARS-Cov-2 infection in PLWHIV and/or TBP.
- To determine the risk factors for COVID-19 occurrence among PLWHIV and/or TBP;
- To analyze the perceptions, psychological experiences and treatment trajectory of PLWHIV and/or TBP during the COVID-19 pandemic.

## Methods

### The design and setting of the study

A multidisciplinary study is being conducted from 2021 to 2023 in Bobo-Dioulasso, Burkina Faso using mixed methods, a combination of quantitative and qualitative methods.

### Phase 1: Sero-epidemiological and clinical study

This component will represent an ambispective study consisting of a retrospective analysis of the circulation of SARS-CoV-2 in patients living with HIV and followed up between March 2020 and March 2021, and a prospective analysis of two cohorts of patients (a cohort of PLWHIV and another cohort of TBP in 2022) for a one-year follow-up period.

### Circulation of SARS-CoV-2 in PLWH

#### The characteristics of participants

The eligible population for this phase of the study will include all PLWHIV followed up at the Souro Sanou University Hospital Center (SSUHC) in Bobo-Dioulasso, a reference center for the care of people living with HIV in the region and responsible for the follow-up of 42% of [20].

#### Inclusion criteria

The inclusion criteria for the study is as follows:

- All HIV-infected patients followed up at the SSUHC between March 2020 and March 2021.
- Aged 18 years or older.
- Having given their informed consent for the use of their medical records and plasma sample for research purposes.

#### Non‑inclusion criteria

- Inappropriate or unavailable plasma samples.

#### Sample size calculation and sampling

A sample size of 353 patients is needed with an expected SARS-CoV-2 seroprevalence of 50%, precision of ± 5%, alpha risk = 5% and no design effect (Deff = 1) [21]. This number was increased to 420 to take into account any unused or unpreserved plasma. All PLWHIV followed up at the SSUHC are registered in a patient database named ESOPE (Evaluation et Suivi Opérationnel des Programmes Esther”) with a unique identifier. Only one sample will be randomly selected for a patient with more than one blood samples.

#### Data collection and processing

The socio-demographic, clinical (age, sex, comorbidities, clinical stage) and biological (Viral Load, CD4 count) data of the selected patients at the follow-up visit during which the blood sample was taken will be retrieved from the patient database. The plasma of HIV-infected patients followed up routinely is kept at -80 °C in a protected biobank at the SSUHC laboratory after seeking the informed consent of the patients. The Enzyme-linked Immunosorbent Assay (ELISA) will be conducted for qualitative detection of total antibodies (IgG) against SARS-CoV-2 in selected samples) [22]. The result of this analysis will be used to complete the database.

#### Study endpoints

The primary endpoint will be the proportion of PLWHIV with specific antibodies to SARS-CoV-2.

The secondary endpoints will be as follows:

- The proportion of HIV-infected persons with specific antibodies to SARS-CoV-2 in each socio-demographic group (age, sex).
- The proportion of HIV-infected persons with specific antibodies to SARS-CoV-2 in each clinical/biological group (clinical stage, CD4 count, Viral Load).

#### Statistical analyses

The following analyses will be performed:

- Calculation of prevalence: the proportion of HIV-positive persons infected with SARS-CoV-2.
- Description and comparison of sociodemographic, clinical and biological characteristics of all patients at the time of sampling according to their SARS-CoV-2 serological status.

Quantitative variables will be described with the mean ± standard deviation and compared using Student’s either t-test or Mann-Whitney U-test. Categorical variables will be described as numbers and percentages and compared using either Pearson’s Chi-square test or Fisher’s exact test. All analyses in this section will be conducted using R 4.2.0 (www.r-project.org).

### Cohorts of PLWHIV and TBP

This will involve the design of two longitudinal cohort studies including the cohort of PLWHIV and TB patients.

#### Characteristics of the participants

The longitudinal cohort studies will be made up PLWHIV or TBP who followed up in different health facilities in Bobo-Dioulasso, the second most affected city by the pandemic.

#### Inclusion criteria

The criteria for inclusion in the cohorts is as follows.

- Infected with HIV and/or tuberculosis.
- Aged 18 years or older.
- Being followed up in one of the HIV or TB care centers in Bobo-Dioulasso.
- Having provided written informed consent.

#### Non-inclusion criteria

- Patients unable to answer the survey questions.
- Patients under legal guardianship or tutorship or other form of legal protection.

#### Sample size calculation and sampling

With a population of PLWHIV and TBP estimated at 10,205 and 210 respectively, a precision of 5% (alpha error of 0.05) and a confidence level of 95%, the maximum sample size (expected proportion of people with covid-19 of 50%) expected is 371 and 137 [21]. These sample sizes are multiplied by 10% to account for the possibility of blindness. Therefore, a total number of 409 PLWHIV and 150 TBP will be included in this cohort study. This population will be distributed in proportion to the size of their follow-up cohort of people living with HIV in six HIV healthcare centers (three public centers and three associations) and four TB healthcare centers. This will involve systematic random recruitment at each of the sites until the expected number of participants is reached. At inclusion data will be collected at four time points, then one, six and twelve months after inclusion. Each participant will be asked to sign an informed consent form, and to fill in an inclusion (Additional file 1) or follow-up (Additional file 2) questionnaire with their socio-demographic data, co-morbidities, vaccination, symptoms related to SARS-CoV-2 infection and exposure. Biological samples will be collected by trained personnel and at the rate described in the table below (Table 1).

**Table 1:** Specimen collection, examination and follow-up schedule for HIV-infected people and TB-patients.

Specimen	Biological analysis	Day 0	Month 6	Month 12
Nasopharyngeal swab	• Real-Time Quantitative Reverse Transcription Polymerase Chain Reaction	+	+	+
Sputum	• Tuberculosis bacillus test	+	+	+
Dried blood spot	• Luminex	+	+	+
Venous blood (3 ml) on EDTA tube	• Enzyme-Linked Immuno Assay • CD4 count • HIV Viral Load	+	+	+
Blood drop on blade	• Testing for Plasmodium	+		

Participants positive for SARS-CoV-2 at inclusion will be additionally followed up biologically on days 7 and 14 from the date of diagnosis of COVID-19 and alternative samples of saliva, fresh stool and urine will be collected in sterile containers according to the following table (Table 2). These alternative samples will be stored at + 4 °C and sent to the laboratory.

**Table 2:** Specimen collection, examination and follow-up schedule for SARS CoV-2 positive HIV-infected persons or TB patients after Day 0

Specimen	Bioanalysis	Day 7	Day 14	Month 6	Month 12
Nasopharyngeal swab ,urine, stool and saliva samples	• Real-Time Quantitative Reverse Transcription Polymerase Chain Reaction	+	+	+	+
Sputum	• Tuberculosis bacillus test	+	+	+	+
Dried blood spot	• Luminex analysis	+	+	+	+
Venous blood (3 ml) on EDTA tube	• Enzyme-Linked Immuno Assay • CD4 count • HIV Viral Load	+	+	+	+
Blood drop on blade	• Testing for Plasmodium	+	+		

The samples will be transported to the SSUHC laboratory with their appropriate blood sample tracking forms within 6 h after collection. For transport to the laboratory, blood and nasopharyngeal samples will be triple packed in an isothermal container with a cooler to preserve the cold chain. Sputum, blood drops on slides and filter paper will be transported at room temperature. Laboratory procedures involving the handling of blood samples will be performed in a biosafety cabinet level 2.

### Laboratory analysis

#### Real-time quantitative reverse transcription polymerase chain reaction (RT-qPCR)

Detection of SARS-CoV-2 in nasopharyngeal swabs will be performed using an RT-qPCR test. A commercially available kit, the Abbott RealTime SARS-CoV-2 kit, will be used according to the manufacturer’s [23]. This kit uses an N-gene test and the RdRp gene to detect SARS-CoV-2 cases. Samples which are positive may be subjected to high throughput sequencing for phylogenetic analysis of circulating variants.

Sequencing is carried out at the reference laboratory for antimicrobial resistance of SSUHC using Oxford Nanopore Technology. The ARTIC Network bioinformatics protocol [24] was followed for whole genome assembly and variant calling steps. A minimum of 20 times depth of coverage was considered for confident base call as per the ARTIC Network guidelines. Below this threshold, the bases were represented with an N character, symbolizing unknown nucleotide base at this position. Genomes with more than 50% coverage were deposited in GISAID database.

#### SARS Cov 2 serological testing

Plasma or serum samples will be obtained after centrifugation and aliquoted within 12 h following blood collection. Otherwise, the blood samples will be temporarily stored at + 4 °C for up to 72 h. Two (2) aliquots of 1 ml of plasma or serum each will be made to minimize the number of freeze-thaw cycles. One of the aliquots will be used for realization of anti-SARS-CoV-2 IgG ELISA assay. The second aliquot will be stored at -80 °C in a protected biobank for quality control, further testing or future research. The ELISA kit S-2442/1.1 “DS-EIA-ANTI-SARS-CoV-2-G(S)” will be used for this experiment.

#### Luminex analysis

This experiment will be performed to detect antibodies specific to COVID-19. This assay will combine different SARS-CoV-2 proteins (i.e., spiked (S), including truncated variants (RBD region) and nucleocapsid structural proteins (N)) and will analyze different classes of immunoglobulins including IgA, IgM and IgG. The ratio of these antibody isotypes will be used to assess the exposure phase of the infection. In addition, several seasonal coronaviruses (alpha and beta coronaviruses) and other pathogens causing respiratory infections will be included in the assay to establish assay parameters, including antigen cross-reactivity and specificity of detection of SARS-CoV-2. All SARS-CoV-2 proteins used in the development of the assay will be derived from baculovirus and mammalian expression platforms as described elsewhere [25].

#### Testing for tuberculous bacillus

Sputum will be spread on a slide (smear) and then stained using Ziehl-Neelsen’s staining technique. The stained smear will be examined under a microscope at x100 magnification. A sputum sample will be decontaminated with NaOH/KOH, liquefied with phosphate buffer. It will then be centrifuged and the pellet will be used for culture.

#### CD4 count

The CD4 LT count is performed on the whole blood sample using the BD Facs Count instrument. The BD FACSPresto™ near-patient CD4 counter includes the instrument, integrated software, sample incubation work station, and single-use disposable cartridges. The system is designed to generate the absolute percentage of CD4 T lymphocytes and total hemoglobin (Hb) concentration in whole blood samples. It uses the flow cytometry technique. When whole blood is added to the reagent tube, fluorochrome-labelled antibodies in the reagents bind specifically to white blood cell surface antigens and a fluorescent nuclear dye binds to nucleated blood cells. After addition of a fixative solution, the sample is analyzed on the BD FACS Count instrument. During sample acquisition, the cells pass through the laser light, causing the labelled cells to fluoresce.

#### HIV viral load

Quantification of plasma HIV viral load was performed using the Abbott m2000 system. This technique is performed in two steps:

- Amplification and detection with the m2000rt using the Abbott Real Time HIV-1® kit.
- Automated extraction with m2000sp using the mSample Preparation Systems RNA kit.

#### Plasmodium testing by thick blood film

This is a manual concentration technique without spreading, which uses twice the volume of blood as the smear (0.25 µL if 300 fields are read) and increases the amount of blood examined by a factor of 20 to 30. The technique enhances the analytical sensitivity of the microscopy examination (classically, a threshold of 10 to 20 parasites / µL), which is important if the parasitemia is low. The blood obtained by capillary sampling is place on a slide and defibrinated by applying circular movements for 2 min. The slide is allowed to dried, dehaemoglobinize. Subsequently, the slide is stained and dried. Parasites (schizonts, trophozoites, gametocytes) are counted using leukocyte cells as a reference (i.e. 100, 200 or 500 leukocytes counted) and with the number of parasites/µL as the unit.

#### Research and development of innovative biomarkers for diagnosis and prognosis

The aim is to measure cytokines and chemokines using the Human Cytokine Standard 27-Plex Assays panel (biorad) on the Bio-Plex 200 system (Bio-Rad, Hercules, CA, USA) in order to explore the influence of SARS-CoV-2 infection on cytokine secretion. Peripheral blood immunological indicators such as Natural Killers (NK), CD4 + T, CD8 + T, Regulatory T Cells (Tregs) and B cells, the expression of cell surface markers as well as the expression of Interferon -γ by CD4 + T, CD8 + T and NK cells will be studied using flow cytometry (BD FACS Canto II) and analyzed with the software (BD FACS Diva).

All epidemiological, clinical and biological data recorded in an electronic case report form will be secured using personal access codes for each researcher with daily synchronization on a secure online server. Real-time checks will be performed to verify the quality of the data. The database will then be exported for statistical analysis.

#### Study endpoints

The primary endpoint will be the proportion of vulnerable people (PLWHIV and TBP) with COVID-19.

The secondary endpoints will be the following:

- The proportion of susceptible individuals (HIV and TB) with specific antibodies against SARS-CoV-2.
- The dynamics of viral load and antibodies against SARS-CoV-2.
- The proportion of SARS-CoV-2 variants and novel biomarkers circulating in HIV and TB patients.
- The average duration of clinical signs in COVID-19.
- Infectivity and duration of SARS-CoV-2 in faeces.

#### Statistical analysis

The following analyses will be performed:

- Calculation of the cumulative incidence: the proportion of vulnerable persons (PLWHIV and TBP) who are seropositive for SARS-CoV-2 infection.
- Description and comparison of socio-demographic, clinical and biological characteristics according to the serological status of the population.
- Identification of SARS-COV-2 variants circulating in PLWHIV and TBP.
- Identification of specific biological markers of SARS-Cov-2 infection in PLWHIV and TBP.
- Characterization of viral load and anti-SARS-CoV-2 antibody dynamics.
- Monitoring the duration and infectivity of viral shedding in urine, stool, saliva.

Quantitative variables will be described as mean ± standard deviation and compared using either Student’s t-test or Mann-Whitney U-test. Categorical variables will be described as numbers and percentages and compared using either the chi-square or Fisher’s exact test. Risk factors for SARS-COV-2 infection in PLWH or TBP will be identified through logistic regression analysis.

### Phase 2: Socio-anthropological study

This phase of the study aims to identify the perceptions, community experiences and response strategies developed for PLWHIV and TBP in the health emergency situation imposed by COVID-19 pandemic. A qualitative empirical study [26] will be conducted in the city of Bobo-Dioulasso over a period of six months to:

- determine the level of knowledge of COVID 19 pandemic and the associated representations;
- define the conditions for trust in intervention strategies for so-called vulnerable people;
- identify the level of reference to conventional care after COVID 19;
- identify the therapeutic response strategies and the reasons for these choices;
- assess the level of compatibility of these response strategies on their health status;
- identify community perceptions about people with HIV/TB and COVID co-infection.

#### Analysis design

Our analysis scheme mainly refers to qualitative methods including strategic and systemic analyses by taking into account the social, economic, cultural and symbolic variables related to the situation of vulnerability used as an argument for the orientation of the therapeutic choices for COVID 19.

#### Participant characteristics

The selection of participants will be based on the criterion of diversification. This principle will make it possible to include in the sample the greatest possible variety of individuals, regardless of their statistical variables (young/old, male/female, educated/illiterate, residents of working-class/residential neighborhoods, history of COVID-19 or not). In addition, the principle of saturation will be adopted and this will mean that the interviewers will stop interviewing a group of respondents each time they feel that they have no more new information at the level of individuals, or at the level of each group surveyed. The sample size is estimated at 110 people, as shown in Table 3. The interviewees are presented in the table below (Table 3). In addition, focus will be carried out with PLWHIV and/or TB former COVID patients.

**Table 3:** Distribution of participants by profile according to the socio-anthropological study

Profile	Number
People living with HIV and former patient COVID 19	10
People living with HIV and current patient COVID 19	5
Tuberculosis and former patient COVID 19	10
Tuberculosis and current patient COVID 19	5
People living with HIV and tuberculosis and former patient COVID 19	10
People living with HIV and tuberculosis and current patient COVID 19	5
Relative of people living with HIV and tuberculosis and former patient COVID 19	5
Relative of people living with HIV and tuberculosis current patient COVID 19	5
General population	10
Health workers	10
Traditherapists	6
Street drug sellers	5
Resource persons	10

#### Data collection and management

This will consist of semi-structured individual interviews, focus groups and direct observation (Additional file 3). The interviews will be recorded and transcribed to represent the corpus of analysis.

#### Statistical analysis

A content analysis of all the interviewees’ speeches will be conducted. The analysis will consist of relating the variables, comparing the results of the observation with the results expected by the hypotheses and interpreting the differences. Data will be converted and analyzed using QDA Miner software.

## Discussion

### Benefits and risks for participants

The collection of samples in this study poses minimal risk to participants. Blood sampling may cause some transient physical and/or psychological discomfort to participants, but does not represent any significant long-term risk. Nasopharyngeal sampling may cause irritation and mild discomfort during the procedure.

The direct benefit to the participant in this study is the knowledge of his or her COVID-19 status, follow-up and possible management. Indirectly, the data collected will help to improve and guide activities aimed at understanding the transmission and impact of SARS-CoV-2 in vulnerable populations, and therefore, to take action to prevent the spread of the virus and reduce its impacts in this population.

#### Protection of study researchers

Researchers collecting samples in this study and those conducting laboratory analyses will be provided with personal protective equipment as recommended by the WHO. Socio-anthropometric survey researchers will also wear protective masks and respect social distancing (at least 1 m between people). All persons included in the sero-epidemiological phase of the study, and any personnel involved in the research and tested positive for SARS-CoV-2 during the course of the study, despite protective hygiene measures, will be referred to the care center for appropriate care. The research team will ensure full follow-up of any person infected during the study activities.

#### Stakeholders

To enable the study to be conducted, we have already set up a steering committee and a scientific committee bringing together representatives of the operational structures of the Burkina Faso Ministry of Health (the National Institute of Public Health, the Centre des Opérations de Réponses aux Urgences Sanitaires, the National Tuberculosis Programme, the Ministry of Health, the Ministry of Health and the Ministry of Health), the National Tuberculosis Programme, the Directorate of Biology Laboratories, the Muraz Centre), the Nazi Boni University, the French Embassy and the French Institute for Research and Development, the University Hospital of Montpelier and experts in epidemiology, biology and the study investigators. The members of these committees will make recommendations for the implementation of the study, monitor the study procedures and provide advice on the interpretation of the results that may be useful to policy makers.

## Electronic supplementary material

Below is the link to the electronic supplementary material.

Supplementary Material 1

Supplementary Material 2

Supplementary Material 3

## Data Availability

The data and materials will be made available on request.

## Abbreviations

SSUH: Souro Sanou University Hospital
CD4: Clusters de différenciation 4
COVID-19: The coronavirus disease 2019
DBS: Dried blood spot
EDTA: Ethylenediamine tetraacetic acid
ELISA: Enzyme‑linked immunosorbent assay
HIV: Human immunodeficiency virus
IgG: Immunoglobulin G
IgM: Immunoglobulin M
NK: Natural Killers
PCR: Polymerase Chain Reaction
PLWHIV: People living with HIV
SARS‑CoV‑2: Severe acute respiratory syndrome coronavirus 2
TBP: Tuberculosis patient
TX: Texas
USA: United States of America
VL: Viral load
WHO: World Health Organization.
