# Supplementary material for: Evaluation of the impact of COVID-19 in people coinfected with HIV and/or tuberculosis in low-income countries: study protocol for mixed methods research in Burkina Faso
Source: BMC Infect Dis. 2023 Feb 22;23:108. doi: 10.1186/s12879-023-08076-4 (PMC9944836; doi:10.1186/s12879-023-08076-4)
Supplement: Supplementary file 1 — Supplementary Material 1 [file 12879_2023_8076_MOESM1_ESM.docx]

**Additional file 3a: English translation of the qualitative interview guide: people living with human immunodeficiency virus or infected by tuberculosis infected by COVID-19**

**Evaluation of the impact of COVID-19 in people coinfected with HIV and/or tuberculosis in low-income countries: study protocol for mixed methods research in Burkina Faso**

Age..............................................

Ethnicity......................................

Gender...........................................

Marital status of respondent..............................

Number of children..............................................

Religion.........................................................

Last level of education ......................................

Economic activities: .....................................................................

HIV status...................................................................

Interviewee's initial reference, telephone/address..................................

Interviewer ID and phone: .......................................

Date and time: ........./.........../ 2022

**Theme 1**: **Etiological aspects, knowledge of COVID and associated representation**

1. Is HIV/TB a disease?

2. What do you think about HIV/TB? (Origin: natural, supernatural, cause, severity...)

3. Origin of COVID 19 according to the respondent (where does COVID 19 come from)

4. What are the causes of the COVID 19? (Natural and physical causes attributed to COVID 19)

5. Are there any supernatural causes related to COVID 19? Explain.

6. Describe manifestations of COVID 19.

7. Different local names (or qualifications) of the disease according to the respondent, what is the meaning of this name?

8. Is this disease comparable to a known local pathology? Which one? Explain.

9. Mode of transmission of COVID 19.

10. How many types of COVID 19 are there? Which ones and explain

11. Knowledge and opinion on the new types: their severity, modes of transmission, opinion on the current evolution of the disease

12. Can a mosquito bite transmit the coronavirus? Can one person get it by contamination with blood? How?

13. Can one person be infected by the coronavirus by sharing a meal with an affected person? How?

14. If yes, what are the factors that would increase the risk of transmission?

15. Can one be infected by the coronavirus by working with an infected person?

16. What do you think about having both HIV/TB and COVID 19 at the same time?

17. Is there any special significance to this?

18. Does COVID 19 have any similarities to HIV/TB? Explain. At what level: transmission, contagion, care?

19. Person's experience: what do the terms refer to: emergency, mass priority, isolation, barrier measures, risk, contamination...

20. Are you afraid of coronavirus, HIV/TB? Why or why not?

21. Opinion on the severity of the forms (variants) of COVID 19.

22. What was your feeling at the beginning of COVID 19 (fear, doubt, resistance, indifference) ? Explain.

23. Was there any additional rejection for you? Explain, at what point in the pandemic (beginning, ongoing or current).

24. Opinion on the evolution of the disease: from 2019 to 2022?

25. Opinion on vaccines (did you do it, why?)

**Theme 2: Prevention Strategies / Respondent Perceptions of Covid-19**

1. How did you protect yourself from the COVID 19 (what medications, herbal tea, diet) ?

2. Why did you do this prevention (immune system strengthening? ) Why?

3. What prevention measures do you know about?

4. Are these prevention measures different from those of non-HIV/TB people? Why or why not?

5. How did you learn about these prevention measures? (radio, TV, hospital, association, grin, etc.)

6. What do you think of these prevention measures (necessary? useless? restrictive? dangerous, unbearable? anti-social?) Explain.

7. Opinion on the effectiveness of these official prevention measures in relation to your current status

8. What meaning do you attribute to each measure (please list).

9. How do you feel about compliance with the measures? Were you able to comply with all of them? (List measures complied with by the respondent and their constraints, list measures not complied with by the respondent and their constraints, reasons for compliance or non-compliance, etc.)

10. Did you feel more isolated during this illness? Explain?

11. What is your opinion on the influence of the COVID 19 prevention measures on your activities (economic, cultural, social: weddings, baptisms, funerals, care, etc.)?

12. What is the respondent's opinion about the influence of COVID 19 prevention measures on the maintenance of social ties (support, compassion, assistance, closeness etc.)?

13. Why did you follow these prevention measures (for survival? ...Explain)

**Theme 3: Therapeutic response strategies during COVID 19 and reasons for choices**

1. How did you adapt to the disruptive situation at the beginning of the pandemic?

2. Do you know someone with HIV/TB who died from COVID 19 ? When did this happen?

3. At the beginning of the pandemic were you left out or neglected in (HIV/TB) care? Explain

4. If yes, how were you treated for HIV/TB and HIV/TB-COVID 19? (List official, unofficial, traditional medicines for each disease)

5. Do you know where to go for coronavirus testing?

6. Do you believe in the Covid test result?

7. Do you trust the analysis of the lab results?

8. When a person has Coronavirus disease what medication should they take?

9. Are you aware of any products (formal or informal) used in the care of Covid-19? Which ones? Was it more effective? Why or why not?

10. Describe your care pathway when you had the COVID 19 (give steps and details of the treatment pathway).

**Theme 4: Intervention strategies for "vulnerable" people defining the conditions for trust in these strategies**

1. How did modern health institutions manage HIV/TB-COVID 19 patients at the time of the sudden onset of the disease?

2. What were the previous intervention strategies (how was HIV/TB care provided before or during the COVID 19 period?

3. How is diagnosis done by health care workers (tools, wearing coveralls....)

4. How and where is HIV/TB testing done, COVID 19?

5. How is current HIV/TB management done, COVID 19?

6. Do you feel that the mass (non-HIV/TB population in this case) has been prioritized over you? Explain

7. Were you left out? Why do you say so?

8. What did you do to protect or heal yourself? (Self-medication, traditional care, plants, which ones? prayer, other....)

9. In your facility, is there a psychological follow-up system in case of harm or exposure to COVID 19?

10. If yes, by whom (which team)?

11. If yes, how do you assess the functioning of this psychological follow-up system?

12. What types of measures do these follow-up devices propose to reduce the transmission of Covid-19 to you?

13. What do you think is the most effective measure or combination of measures to reduce transmission of Covid-19 for you?

14. Do you have confidence in the treatment given during the COVID 19 period for you?

15. How do you feel about the effectiveness of the therapies offered when you were infected with COVID 19?

**Theme 5: Level of referral to conventional care after COVID 19/ Perception of procedures related to the FASO-COVID study**

1. Did you attend a center in the early hours of the pandemic? Which one? Which one? Why?

2. What was the management like in the early hours of COVID 19? (Were there any disruptions in HIV/TB prevention? In the regularity of HIV/TB treatment?)

3. How would you rate the management of the health authority?

4. Has this had an effect on your confidence in the health institution? Why

5. Will you continue to follow the prescriptions as in the past? Why or why not? After the break-up period

6. Have you used other types of medicine? If so, was it more effective? Why or why not?

7. Opinion on blood sampling, saliva sampling, nasal sampling (pain, quantity, depth, inconvenience) for the covid test

8. Opinion on home visits (in terms of discretion, discomfort, exposure, time...)

9. Who are the contacts (types of relationships with these people)/what types of relationships do you have with the contacts?

10. Opinion on the time to devote to the study (inclusion date when?, number of months?)

11. Opinion on informed consent for participation in this study, understanding, expectation (Explain)

12. Opinion on study protocols and procedures

13. Notice of financial compensation given (for travel, and communication)

**Theme 6: Level of compatibility of the response strategies adopted during COVID on their HIV/TB situation**

1. What is your relationship with health care staff before COVID 19, during COVID 19, currently?

2. Describe the behavior of health care providers during your care in the early hours of COVID 19?

3. What impact did COVID 19 have on the continuity of your care or management?

4. What is your opinion of health care workers' protective gear to protect themselves from HIV/TB and COVID 19?

5. What do you have to say to improve the relationship with health workers?

6. What is the therapeutic relationship like with other health practitioners (naturopaths, traditherapists...) Explain the process.

7. What is the difference?

**Theme 7: Co-infection situations (HIV/TB+COVID 19) and community perception, or social representations of HIV/TB+COVID 19 co-contamination situations**

1. Family type (monogamous, polygamous, rank in family)

2. Are people in the family aware of your HIV/TB status? Why

3. Have people in the family been aware of your positive COVID 19 result? Why

4. Types of relationship with biological family members (rejection, acceptance, rejection of some people, which ones and why?)

5. Do people in your neighborhood (who) know about your HIV/TB status? Why

6. How did you experience this co-infection? With the family? with others? with health care workers?

7. Were you rejected? Explain people's behavior, tell anecdotes, or stories related to this issue.

8. Opinions on whether the family should protect themselves from HIV/TB and COVID 19 while you are there?

9. Opinions on whether others are careful to protect themselves from HIV/TB and COVID 19 while you are there?

10. Why do you seek treatment (for yourself or to avoid making the community look worse at you or your family)?

11. How do you feel about the current situation of declining Covid?

12. General opinion on this issue of COVID 19 today.

Thank you !

**Additional file 3b: English translation of the qualitative interview guide: general population.**

**Evaluation of the impact of COVID-19 in people coinfected with HIV and/or tuberculosis in low-income countries: study protocol for mixed methods research in Burkina Faso**

Age..............................................

Ethnicity......................................

Gender...........................................

Marital status of respondent..............................

Number of children..............................................

Religion.........................................................

Last level of education ......................................

Economic activities: .....................................................................

HIV status...................................................................

Interviewee's initial reference, telephone/address..................................

Interviewer ID and phone: .......................................

Date and time: ........./.........../ 2022

**Theme 1: Disease knowledge/ Representation system/ Perception**

1. Do you have a relative or acquaintance who has HIV or TB? (Name in language, meaning of name), Who? Relationship?

2. What does it mean when someone has this disease?

3. What are the causes, symptoms?

4. What are the manifestations?

5. Methods of contamination (how can one get the disease?)

6. Do you know the disease that people call COVID 19? Name in your country, meaning

7. Do you know anyone with HIV or TB who has had it, what do you think?

8. What were the manifestations on their condition?

9. Are there any similarities between COVID 19 and HIV/TB?

10. In addition to modern drugs, do these people with HIV or TB use other treatments? Which ones (which local plants, traditional therapists, prayers, market medicines...)?

11. When a person has HIV/TB/COVID 19 , what is your attitude towards that person? Why or why not?

12. Is this person even more "contaminating" than others who do not have HIV/TB? Explain

**Theme 2: Social Approach**

1. In your opinion, do family members hide their HIV- or TB-infected relative? Why or why not?

2. Can you become infected with the coronavirus, TB/HIV by sharing a meal with someone who is sick?

3. Can you become infected with the TB/HIV virus by living in the same house with someone who has the disease?

4. Can you become infected with the TB/HIV virus by working with someone who has the disease? (If so, what factors would increase the risk of transmission?)

5. Can you become infected with the TB/HIV virus by traveling with someone who has the disease?

6. What would you do to help these HIV/TB people cope with their condition?

7. Covid's opinion on this issue today

Thank you very much

**Additional file 3c: English translation of the qualitative interview guide: health workers**

**Evaluation of the impact of COVID-19 in people coinfected with HIV and/or tuberculosis in low-income countries: study protocol for mixed methods research in Burkina Faso**

Age..............................................

Ethnicity......................................

Gender...........................................

Marital status of respondent..............................

Number of children..............................................

Religion.........................................................

Last level of education ......................................

Economic activities: .....................................................................

HIV status...................................................................

Interviewee's initial reference, telephone/address..................................

Interviewer ID and phone: .......................................

Date and time: ........./.........../ 2022

**Theme 1: Sources of Information, Views of Disease and Management Covid-19**

1. How many years have you worked as a health worker at this facility (total time in practice)?

2. In what position do you work? (Multiple positions are possible)

3. Do you work in another health facility? Which one?

4. What is your main source of information about COVID 19?

5. In the early days of COVID 19, were there any difficulties in the supply chain of drugs for HIV/TB management? Explain

6. Were there any shortages of products needed for the management of PLWHA? Explain

7. What were the alternatives?

8. What did patients do during these times of uncertainty?

9. How many HIV/TB+ patients do you have in COVID 19?

10. In the early hours of COVID 19, what did you observe (regarding attendance...) of PLWHIV in the centers? What were their attitudes?

**Theme 2: Prevention and Care**

1. What prevention strategies were advocated or dictated for these particular HIV/TB individuals?

2. How was prevention for PLWHIV or TB done before COVID 19, in the early days of COVID 19?

3. How is HIV/TB/COVID 19 prevention done now?

4. How was the management of PLWHIV/TB done before, in the early hours and currently?

5. What are the effects of COVID 19 on the health status of TB/HIV patients?

6. What care strategies were dictated for these HIV/TB individuals?

7. How many people routinely ask you for a diagnostic test at COVID 19 ?

8. How many HIV/TB people routinely ask you for a vaccine?

9. Have you ever been tested/vaccinated (CORONA)? Are you currently willing to do so? Why or why not?

10. When a person has Coronavirus disease where should they be treated?

11. What diagnostic methods do you use?

12. How does it work (difficulties of handling, technique of use?...)

13. What is the profile of the personnel designated to take care of COVID 19 patients? Does this require any special skills?

14. Are there any points to improve in the system? Which ones?

15. Opinion on the method used (accuracy, time, reliability, speed) in relation to the diagnosis

16. When is Corona testing recommended for PLWH/TB?

17. What is the protocol for positive diagnoses? What is the protocol (other treatment, new diagnosis)

18. In retrospect, what is your opinion of this arrangement?

**Theme 3: Medical-psychological follow-up**

1. In your facility, is there a medical follow-up system for PLWHA/TB exposed to Covid-19? How does this system work?

2. Does your facility have a psychological follow-up system for PLWHA/TB exposed to Covid-19?

3. What are the skills that make up the team?

4. How do you feel about the functioning of the psychological follow-up system?

5. In your opinion, if a health worker or other health care personnel is infected with the coronavirus (but does not have the signs) should they be allowed to continue practicing?

6. In your opinion, if someone close to you is infected with coronavirus (tested positive without signs) or sick (tested positive with signs), would you be willing to care for them at home?

**Theme 4: Therapeutic cooperation: traditional medicine (Traditherapy) and modern medicine**

1. What types of relationships do you have with traditional medicine practitioners?

2. Do you have any information regarding the simultaneous use of both medicines (modern and traditional) by PLWHA? Motivation?

3. Do you have any information regarding the simultaneous use of both or more medicines (modern and traditional...) by PLWHA/TB during the COVID 19 period? Why?

4. Aspects related to the degree of confidence in these alternative therapies, Explain

5. What lessons have you learned as a practitioner from this pandemic?

Thank you!

**Additional file 3d: English translation of the qualitative interview guide: traditional/naturo therapists**

**Evaluation of the impact of COVID-19 in people coinfected with HIV and/or tuberculosis in low-income countries: study protocol for mixed methods research in Burkina Faso**

Neighborhood: ..............................................................................

Marital status of respondent..............................

Religion.........................................................

Last grade completed ......................................

Age..............................................

Ethnicity......................................

Gender...........................................

Other economic activities: .....................................................................

Telephone/address of Tradithérapeute ..................................

Investigator ID: .......................................

Date: ........./.........../2022

Time of survey: ...........

**Theme 1: Knowledge about the disease/treatment procedures**

1. Modalities of knowledge acquisition: Inheritance -Dream -Revelation-Learning- Gift- Initiating disease- Other...

2. What are the diseases for which you are solicited? Reasons

3. Do you know HIV? TB? COVID 19? the name of these diseases in your language? Manifestation? Symptoms?

4. Do you treat HIV? TB? How and with what medication?

5. Are these natural, supernatural, or other diseases? Why or why not?

6. At what stage of the disease (HIV/TB) are you being asked? Why?

7. What does the HIV/TB diagnosis and treatment consist of (therapeutic procedure, speech,...),

8. What is the COVID 19 HIV/TB diagnosis and treatment?

9. Are there several types of these diseases? Which ones?

10. Do the treatments address the different types identified?

11. Practices used in the treatments: Modern medicine, Herbs, Rites, Powders, Other...

12. Do HIV/TB/COVID 19 have similarities between them? Or are they different diseases? Explain

13. Are there similarities with other diseases? What are they? Explain

14. Is it more difficult to treat this co-contamination (HIV/TB/COVID 19)? Why or why not?

**Theme 2: Traditional Caregiver-Patient Relationship**

1. Existence of cultural proximity (place of kinship) in traditional medicine

**Theme 3: Cooperation (professional relationship) between modern medicine and traditional medicine**

1. Type of relationship with health workers in modern medicine (doctors, nurses, administrative staff, other practitioners, etc.) during the COVID 19 period.

2. Do you receive patients from home?

3. Do you see patients with HIV/TB+COVID 19?

4. Do you see HIV/TB+COVID 19 patients who have previously seen health workers? Why do they come to you afterwards?

5. Do you use the results of modern medicine diagnoses for further treatment? Why?

6. Are there any conflicts between you and the agents of modern medicine (of power, political relations between the different components of the actors)?

7. What do you think about this issue of Covid 19 today?

I thank you!

**Additional file 3e: English translation of the qualitative interview guide: street drug sellers**

**Evaluation of the impact of COVID-19 in people coinfected with HIV and/or tuberculosis in low-income countries: study protocol for mixed methods research in Burkina Faso**

Neighborhood: ..............................................................................

Marital status of respondent..............................

Religion.........................................................

Last grade completed ......................................

Age..............................................

Ethnicity......................................

Gender...........................................

Economic activities: .....................................................................

Vendor phone/address ..................................

Investigator ID: .......................................

Date: ........./.........../2022 Time :

**Theme 1: Disease Knowledge/Therapeutic Procedures**.

1. What diseases are you being asked to treat? Reasons

2. Are you familiar with HIV? TB? Symptoms?

3. Do you treat HIV? TB? With which drugs (name), origins of these drugs

4. Is it a natural disease, a supernatural disease or Other..................

5. At what stage of the disease (HIV/TB) are you being asked? Reasons?

6. Do you make diagnoses? How do you do it?

7. How is the HIV/TB treatment done (therapeutic procedure, speech,...),

8. What is the COVID 19 HIV/TB diagnosis and treatment?

9. What are the symptoms on which you base your HIV/TB diagnosis?

10. Are there different types of these diseases? What are they?

11. Do the treatments address the different types identified?

12. Practices used in treatments: Types of Medication (names on boxes and local names, photos.... Other...)

13. Do HIV/TB/COVID 19 have similarities between them? Or are they different diseases? Explain

14. They have similarities with which diseases, explain

15. Is it more difficult to treat this co-infection (HIV/TB/COVID 19)? Why or why not?

**Theme 2: Caregiver-patient relationship**

1. Do you know the patients (link? Meeting place for care......)

2. Existence of cultural proximity (place of relatives) in self-medication

3. What explains this confidence in your medication?

Theme 3: Perception of street medicines

1. Type of relationship with modern security agents (police, town hall, other ...) during the COVID 19 period.

2. Where do you receive patients from their homes? in the management of HIV/TB/COVID 19 ?

3. Do you see patients who have previously consulted health workers? Why do they come to you afterwards?

4. Do you use the results of modern medicine diagnoses for further treatment? Why?

5. Are there any health hazards associated with the use of your medications? Explain

6. What do you think about this question from Covid today?

Thank you!

**Additional file 3f: English translation of the qualitative interview guide: resource persons**

**Evaluation of the impact of COVID-19 in people coinfected with HIV and/or tuberculosis in low-income countries: study protocol for mixed methods research in Burkina Faso**

**The HIV and TB Program Manager**

1. Introduce yourself,

2. What are your missions?

3. What was your role before the COVID 19 period?

4. What is your role and contribution during the pandemic?

5. What is your role and contribution now?

6. Has COVID 19 disrupted the supply chain for products needed in HIV/TB prevention and care? Explain

7. What are the main difficulties in carrying out your missions during the health crisis?

8. What do you think of the management of the pandemic in BF?

9. Have you been vaccinated? Why or why not?

10. What do you think about vaccination? Of the types offered at the BF?

11. What needs to be done to improve prevention and care?

**An official from the Regional Health Department**

1. Introduce yourself,

2. What are your missions?

3. What was your role before the COVID 19 period?

4. What is your role and contribution during the pandemic?

5. What is your role and contribution now?

6. Has COVID 19 disrupted the supply chain for products needed in HIV/TB prevention and care? Explain

7. What are the main difficulties in carrying out your missions during the health crisis?

8. What do you think of the management of the pandemic in BF?

9. Have you been vaccinated? Why or why not?

10. What do you think about vaccination? Of the types offered at the BF?

11. What needs to be done to improve prevention and care?

**The Governor (Chair of the Regional Health Committee)**

1. Introduce yourself,

2. What are your responsibilities?

3. What was your role and contribution before the COVID 19 period?

4. What is your role and contribution during the pandemic?

5. What is your role and contribution now?

6. Has COVID 19 disrupted the supply chain for products needed in HIV/TB prevention and care? Explain

7. What are the main difficulties in carrying out your missions during the health crisis?

8. What do you think of the management of the pandemic in BF?

9. Have you been vaccinated? Why or why not?

10. What do you think about vaccination? Of the types offered at the BF?

11. What should be done to improve prevention and care?

**The Chair of the HIV Committee**

1. Introduce yourself,

2. What are your missions?

3. What was your role and contribution before the COVID period?

4. What is your role and contribution during the pandemic?

5. What is your role and contribution now?

6. Has COVID 19 disrupted the supply chain for products needed in HIV/TB prevention and care? Explain

7. What are the main difficulties in carrying out your missions during the health crisis?

8. What do you think of the management of the pandemic in BF?

9. Have you been vaccinated? Why or why not?

10. What do you think about vaccination? Of the types offered at the BF?

11. What should be done to improve prevention and care?

**Opinion leaders (from the community sector)**

1. Introduce yourself,

2. What are your missions?

3. What was your role and contribution before the COVID 19 period?

4. What is your role and contribution during the pandemic?

5. What is your role and contribution now?

6. What do you think about HIV/TB? Explain (natural, unnatural disease?) Explain

7. Do you have cases of co-infection (PLWHIV/TB/COVID 19)? How was the management done?

8. Did you receive support? Which institution? What equipment?

9. What were the main difficulties in carrying out your missions during the health crisis?

10. What do you think of the management of the pandemic in BF?

11. Have you been vaccinated? Why or why not?

12. What do you think about vaccination? The types of vaccines offered at the BF?

13. What needs to be done to improve prevention and care?
