# Supplementary material for: Evaluation of the impact of COVID-19 in people coinfected with HIV and/or tuberculosis in low-income countries: study protocol for mixed methods research in Burkina Faso
Source: BMC Infect Dis. 2023 Feb 22;23:108. doi: 10.1186/s12879-023-08076-4 (PMC9944836; doi:10.1186/s12879-023-08076-4)
Supplement: Supplementary file 2 — Supplementary Material 2 [file 12879_2023_8076_MOESM2_ESM.docx]

**Evaluation of the impact of COVID-19 in people coinfected with HIV and/or tuberculosis in low-income countries: study protocol for mixed methods research in Burkina Faso: People living with HIV follow form**

**GENERAL INFORMATION**

1. Follow-up period: (1=M1; 2=M6; 3=M12) ? |__|

2. Date of follow-up: __ __/__ __/__ __ __ __

3. Full name of investigator ...........................................................................

4. Participant ID: |__||__|

**UNDERLYING CONDITIONS AND COMORBIDITIES**

5. Pregnancy (0=No; 1=Yes; 2=NA) ?

6. If yes, specify the quarter: |__| (1,2,3 or 4)

7. Postpartum (0=No; 1=Yes; 2=NA) ?

8. Diabetes (0=No ; 1=Yes ) ? |__|

9. Liver disease (0=No ; 1=Yes ) ? |__|

10. If yes, specify the disease: _____________________

11. Kidney disease (0=No ; 1=Yes ) ? |__|

12. If yes, specify the disease: _____________________

13. Cardiovascular disease including known and treated hypertension (0=No ; 1=Yes )? |__|

14. If yes, specify the disease: _____________________

15. Chronic neurological or neuromuscular disease (0=No ; 1=Yes )? |__|

16. If yes, specify the disease: _____________________

17. Chronic lung disease other than tuberculosis (0=No; 1=Yes)? |__|

18. If yes, specify the disease: _____________________

19. Smoking (0=No; 1=Yes) ?

20. If yes, specify the number of packages/year |__||__|

21. Cancer (0=No ; 1=Yes ) ? |__|

22. If yes, specify the organ affected: _____________________

23. Other comorbidities, please specify: __________________________________________

24. Have you tested positive for Covid-19 since your last visit (0=No; 1=Yes)? |__|

25. Have you had a Covid-19 vaccination since your last visit? (0=No; 1=Yes) |__|

26. If Yes, which vaccine 1 (1=AstraZeneca; 2=Johnson and Johnson; 3=Sinopharm; 4=Pfizer, 5=Other)? |__|

27. Si autre préciser_____________________________________________

28. Number of doses |__||__|

29. Date of Dose 1 Date :____ /___/____/202__ or |__||__||__|| weeks

30. Date of Dose 2 Date :____ /___/____/202__ or |__||__|||__| weeks

31. If Yes, which vaccine 2 (1=AstraZeneca; 2=Johnson and Johnson; 3=Sinopharm; 4=Pfizer,

5=Other)? |__|

32. Si autre préciser_____________________________________________

33. Number of doses |__|||__|

34. Date of Dose 1 Date :____ /___/____/202__ or |__||__||__|| weeks

35. Date of Dose 2 Date :____ /___/____/202__ or |__||__|||__| weeks

**STATUS TRACKING :**

36. Are you on ARV treatment (0=No; 1=Yes)?

37. How long have you been on ARV treatment: Date:____ /___/____/202__

38. What is your initial treatment regimen (1=TLD(TDF/3TC/DTG); 2=TLE(TDF/3TC/EFV);

3=ABC/3TC/DTG; 4= ABC/3TC/EFV; 5=Other)? |__|

39. If other, please specify_________________________________________

40. Have you ever changed your ARV treatment (0=No; 1=Yes)? |__|

41. If yes, what is your current treatment regimen (1=TLD(TDF/3TC/DTG); 2=TLE(TDF/3TC/EFV);

3=ABC/3TC/DTG; 4= ABC/3TC/EFV; 5=Other)? |__|

42. If other, please specify_________________________________________

43. How old is it? Date:____ /___/____/202__

44. Are you on Chemoprophylaxis (0=No; 1=Yes)?

45. If yes, which ones (1=Cotrimoxazole ; 2=Isoniazide ; 3=Other) If Other, please specify

__________________

46. Are you/have you been on treatment for any opportunistic infections (0=No; 1=Yes)?

If yes which one(s) :

47. Tuberculosis (0=No; 1=Yes) ?

48. Toxoplasmosis (0=No; 1=Yes) ?

49. Prurigo (0=No; 1=Yes) ?

50. Digestive candidiasis (0=No; 1=Yes) ?

51. Isosporosis (0=No; 1=Yes) ?

52. Cryptococcosis (0=No; 1=Yes) ?

53. Autre(s), préciser_________________________________________________________________

54. When was your last viral load: Date:____ /___/____/202__

55. What was your last viral load result (0=Undetectable; 1=Detectable)? |__|

56. If detectable, specify the number : __________________ __________________

57. When was your last CD4 count: Date:____ /___/____/202__

58. What was your last CD4 count __________________

59. What is the participant's current WHO stage (1=Stage 1; 2=Stage 2; 3=Stage 3; 4=Stage 4)? |__|

**If HIV-TB CO-INFECTION**

60. Are you on anti-tuberculosis treatment (0=No; 1=Yes)? |__|

61. How long have you been on treatment : Date:____ /___/____/202__

62. What is your initial treatment regimen __________________

63. Have you ever changed your TB treatment (0=No; 1=Yes)?

64. If yes, what is your current treatment regimen __________________

65. How old is it? Date:____ /___/____/202__

66. When was your last bacilloscopy: Date:____ /___/____/202__

67. What was the result of your last bacilloscopy (0=Negative; 1=Positive)? |__|

68. When was your last Xpert MTB/RIF test Date:____ /___/____/202__

69. What was the result of this Xpert MTB/RIF test: (0=Negative; 1=Positive)?

**CLINICAL INFORMATION**

70. Do you have any symptoms (0=No; 1=Yes)? |__|

71. If yes, since when: Date:____ /___/____/202__

72. If yes, specify symptoms _____ __________________________________

What are the participant's constants:

73. Temperature(°C) : |__||__| , |__|

74. Weight(kg) : |__||__|||

75. Size(cm) : |__||__|||

76. Respiratory rate (cycles/min): |__||__|

77. Sap02(%) : |__||__|

78. Pulse (beats/min): |__||__||

**POTENTIAL EXPOSURE TO COVID 19 IN THE LAST 14 DAYS (OR PRIOR TO SYMPTOM**

**ONSET)**

79. Have you traveled in the last 14 days (0=No; 1=Yes)? |__|

80. If yes, please specify where ........................

81. When? __ __/__ __/__ __; or approximate duration in days |__||

82. Have you been in contact with a confirmed or probable COVID-19 case in the past 14 days (0=No;

1=Yes)? |__|

83. If yes. Specify: The nature of the relationship with the contact (no name)....................................

84. Place of contact? ......... .........When? __ __/_ __/____; or the approximate duration in days |__|

85. Have you attended a mass gathering (e.g., wedding, baptism, market) in the past 14 days (0=No;

1=Yes)? |__|

86. If yes, please specify which one? ................................Location?..................When? __ __/__ __/__

__

or the approximate duration in days |__|__|

87. Have you visited a health facility/traditional healer in the past 14 days (as a patient/attendant)?

(0=No; 1=Yes) |__|

88. If yes, please specify: Which institution?..................Location?...............................

89. When? __ __/__ __/__ __ __ __ ; or approximate duration in days |__||

90. Do you live in contact with animals? (0=No; 1=Yes)?

91. |__| if yes which ones:.............................

92. Have you visited a live animal market in the past 14 days (0=No; 1=Yes)? |__|

93. Have you regularly worn a mask in public places in the past 14 days? (0=No; 1=Yes) |__|

94. Have you regularly observed hand hygiene in the past 14 days (0=No; 1=Yes)? |__|

95. Have you regularly observed the distancing measures in public during the past 14 days (0=No;

1=Yes)? |__|

**PARACLINICAL EXAMINATIONS**

96. Was a nasopharyngeal sample taken (0=No; 1=Yes)?

97. Was sputum with induced sputum collected (0=No; 1=Yes)?

98. Was venous blood 1 drawn (0=No; 1=Yes)? |__|

99. Was venous blood 2 drawn (0=No; 1=Yes)?

100. Was a drop of dried blood collected on filter paper (0=No; 1=Yes)? |__|

101. Was a thick drop performed (0=No; 1=Yes)? |__|

102. Was a saliva sample taken (0=No; 1=Yes)?

103. Was a urine sample taken (0=No; 1=Yes)?

104. Was a stool sample taken (0=No; 1=Yes)?

**Evaluation of the impact of COVID-19 in people coinfected with HIV and/or tuberculosis in low-income countries: study protocol for mixed methods research in Burkina Faso: Patients with tuberculosis follow form**

**GENERAL INFORMATION**

1. Follow-up period: (1=M1; 2=M6; 3=M12) ? |__|

2. Date of follow-up: __ __/__ __/__ __ __ __

3. Full name of investigator ...........................................................................

4. Participant ID: |__||__|||

**UNDERLYING CONDITIONS AND COMORBIDITIES DURING FOLLOW-UP**

5. Pregnancy (0=No; 1=Yes; 2=NA) ?

6. If yes, specify the quarter: |__| (1,2,3 or 4)

7. Postpartum (0=No; 1=Yes; 2=NA) ?

8. Diabetes (0=No ; 1=Yes ) ? |__|

9. Liver disease (0=No ; 1=Yes ) ? |__|

10. If yes, specify the disease: _____________________

11. Kidney disease (0=No ; 1=Yes ) ? |__|

12. If yes, specify the disease: _____________________

13. Cardiovascular disease including known and treated hypertension (0=No ; 1=Yes )? |__|

14. If yes, specify the disease: _____________________

15. Chronic neurological or neuromuscular disease (0=No ; 1=Yes )? |__|

16. If yes, specify the disease: _____________________

17. Chronic lung disease other than tuberculosis (0=No; 1=Yes)? |__|

18. If yes, specify the disease: _____________________

19. Smoking (0=No; 1=Yes) ?

20. If yes, specify the number of packages/year |__||__|||

21. Cancer (0=No ; 1=Yes ) ? |__|

22. If yes, specify the organ affected: _____________________

23. HIV infection (0=No; 1=Yes) ?

24. Other comorbidities, please specify: ___________________________________________

25. Have you tested positive for Covid-19 since your last visit (0=No; 1=Yes)? |__|

26. Have you had a Covid-19 vaccination since your last visit? (0=No; 1=Yes) |__|

27. If Yes, which vaccine 1 (1=AstraZeneca; 2=Johnson and Johnson; 3=Sinopharm; 4=Pfizer,

5=Other)? |__|

28. Si autre préciser_____________________________________________

29. Number of doses |__|||__|

30. Date of Dose 1 Date :____ /___/____/202__ or |__||__||__|| weeks

31. Date of Dose 2 Date :____ /___/____/202__ or |__||__|||__| weeks

32. If Yes, which vaccine 2 (1=AstraZeneca; 2=Johnson and Johnson; 3=Sinopharm; 4=Pfizer,

5=Other)? |__|

33. Si autre préciser_____________________________________________

34. Number of doses |__|||__|

35. Date of Dose 1 Date :____ /___/____/202__ or |__||__||__|| weeks

36. Date of Dose 2 Date :____ /___/____/202__ or |__||__|||__| weeks

**STATUS TRACKING :**

Are you on anti-tuberculosis treatment (0=No; 1=Yes)? |__|

How long have you been on treatment : Date:____ /___/____/202__

What is your initial treatment regimen __________________

Have you ever changed your TB treatment (0=No; 1=Yes)?

If yes, what is your current treatment regimen __________________

How old is it? Date:____ /___/____/202__

When was your last bacilloscopy: Date:____ /___/____/202__

What was the result of your last bacilloscopy (0=Negative; 1=Positive)? |__|

When was your last Xpert MTB/RIF test Date:____ /___/____/202__

What was the result of this Xpert MTB/RIF test: (0=Negative; 1=Positive)?

**IF HIV-TB CO-INFECTION**

37. Are you on ARV treatment (0=No; 1=Yes)?

38. How long have you been on ARV treatment: Date:____ /___/____/202__

39. What is your initial treatment regimen (1=TLD(TDF/3TC/DTG); 2=TLE(TDF/3TC/EFV);

3=ABC/3TC/DTG; 4= ABC/3TC/EFV; 5=Other)? |__|

40. If other, please specify_________________________________________

41. Have you ever changed your ARV treatment (0=No; 1=Yes)? |__|

42. If yes, what is your current treatment regimen (1=TLD(TDF/3TC/DTG); 2=TLE(TDF/3TC/EFV);

3=ABC/3TC/DTG; 4= ABC/3TC/EFV; 5=Other)? |__|

43. If other, please specify_________________________________________

44. How old is it? Date:____ /___/____/202__

45. Are you on Chemoprophylaxis (0=No; 1=Yes)?

46. If yes, which ones (1=Cotrimoxazole ; 2=Isoniazide ; 3=Other) If Other, please specify

__________________

47. Are you/have you been on treatment for any opportunistic infections (0=No; 1=Yes)?

If yes which one(s) :

48. Tuberculosis (0=No; 1=Yes) ?

49. Toxoplasmosis (0=No; 1=Yes) ?

50. Prurigo (0=No; 1=Yes) ?

51. Digestive candidiasis (0=No; 1=Yes) ?

52. Isosporosis (0=No; 1=Yes) ?

53. Cryptococcosis (0=No; 1=Yes) ?

54. Autre(s), préciser_________________________________________________________________

55. When was your last viral load: Date:____ /___/____/202__

56. What was your last viral load result (0=Undetectable; 1=Detectable)? |__|

57. If detectable, specify the number : __________________ __________________

58. When was your last CD4 count: Date:____ /___/____/202__

59. What was your last CD4 count __________________

60. What is the participant's current WHO stage (1=Stage 1; 2=Stage 2; 3=Stage 3; 4=Stage 4)? |__|

**CLINICAL INFORMATION**

61. Do you have any symptoms (0=No; 1=Yes)? |__|

62. If yes, since when: Date:____ /___/____/202__

63. If yes, specify symptoms _____ __________________________________

What are the participant's constants:

64. Temperature(°C) : |__||__| , |__|

65. Weight(kg) : |__||__|||

66. Size(cm) : |__||__|||

67. Respiratory rate (cycles/min): |__||__|

68. Sap02(%) : |__||__|

69. Pulse (beats/min): |__||__||

**POTENTIAL EXPOSURE TO COVID 19 IN THE LAST 14 DAYS (OR PRIOR TO SYMPTOM ONSET)**

70. Have you traveled in the last 14 days (0=No; 1=Yes)? |__|

71. If yes, please specify where ........................

72. When? __ __/__ __/__ __; or approximate duration in days |__||

73. Have you been in contact with a confirmed or probable COVID-19 case in the past 14 days (0=No;

1=Yes)? |__|

74. If yes. Specify: The nature of the relationship with the contact (no name)....................................

75. Place of contact? ......... .........When? __ __/_ __/____; or the approximate duration in days |__|

76. Have you attended a mass gathering (e.g., wedding, baptism, market) in the past 14 days (0=No;

1=Yes)? |__|

77. If yes, please specify which one? ................Location ?..................When? __ __/__ __/__

or the approximate duration in days |__|__|

78. Have you visited a health facility/traditional healer in the past 14 days (as a patient/attendant)?

(0=No; 1=Yes) |__|

79. If yes, please specify: Which institution?..................Location?...............................

80. When? __ __/__ __/__ __ __ __ ; or approximate duration in days |__||

81. Do you live in contact with animals? (0=No; 1=Yes)?

82. |__| if yes which ones:.............................

83. Have you visited a live animal market in the past 14 days (0=No; 1=Yes)? |__|

84. Have you regularly worn a mask in public places in the past 14 days? (0=No; 1=Yes) |__|

85. Have you regularly observed hand hygiene in the past 14 days (0=No; 1=Yes)? |__|

86. Have you regularly observed the distancing measures in public during the past 14 days (0=No;

1=Yes)? |__|

**PARACLINICAL EXAMINATIONS**

87. Was a nasopharyngeal sample taken (0=No; 1=Yes)?

88. Was sputum with induced sputum collected (0=No; 1=Yes)?

89. Was venous blood 1 drawn (0=No; 1=Yes)? |__|

90. Was venous blood 2 drawn (0=No; 1=Yes)?

91. Was a drop of dried blood collected on filter paper (0=No; 1=Yes)? |__|

92. Was a thick drop performed (0=No; 1=Yes)? |__|

93. Was a saliva sample taken (0=No; 1=Yes)?

94. Was a urine sample taken (0=No; 1=Yes)?

95. Was a stool sample taken (0=No; 1=Yes)?
