# Supplementary material for: Evaluation of the impact of COVID-19 in people coinfected with HIV and/or tuberculosis in low-income countries: study protocol for mixed methods research in Burkina Faso
Source: BMC Infect Dis. 2023 Feb 22;23:108. doi: 10.1186/s12879-023-08076-4 (PMC9944836; doi:10.1186/s12879-023-08076-4)
Supplement: Supplementary file 3 — Supplementary Material 3 [file 12879_2023_8076_MOESM3_ESM.docx]

**Evaluation of the impact of COVID-19 in people coinfected with HIV and/or tuberculosis in low-income countries: study protocol for mixed methods research in Burkina Faso: People living with HIV inclusion form**

**GENERAL INFORMATION**

1. Date of inclusion: __ __/__ __/2022

2. Name of the investigator .......................................

3. Participant ID: |__||__|||

**SOCIO-DEMOGRAPHIC CHARACTERISTICS**

4. Initial name(s): ___________________________

5. Date of birth: ____/____/______ or Age in years: ____

6. Gender: □ Female □ Male

7. Religion: **(**1=Muslim; 2=Christian; 3=Traditional; 4=Other)? |__| Other religion to specify

__________

8. Occupation (1=Public employee; 2=Private employee; 3=Farmer; 4=Farmer, 5=Trading,

6=Pupil/Student, 7=Housewife, 8=Informal sector, 9=Retired, 10=Other)? |__| Other occupation

to be specified ______________________________

9. District of residence: ______________

10. City/Town: ______________

11. Sector : __________

12. Place of residence: □ Urban / □ Rural

**UNDERLYING CONDITIONS AND COMORBIDITIES**

13. Pregnancy (0=No; 1=Yes; 2=NA) ?

14. If yes, specify the quarter: |__| (1,2,3 or 4)

15. Postpartum (0=No; 1=Yes; 2=NA) ?

16. Diabetes (0=No ; 1=Yes ) ? |__|

17. Liver disease (0=No ; 1=Yes ) ? |__|

18. If yes, specify the disease: _____________________

19. Kidney disease (0=No ; 1=Yes ) ? |__|

20. If yes, specify the disease: _____________________

21. Cardiovascular disease including known and treated hypertension (0=No ; 1=Yes )? |__|

22. If yes, specify the disease: _____________________

23. Chronic neurological or neuromuscular disease (0=No ; 1=Yes )? |__|

24. If yes, specify the disease: _____________________

25. Chronic lung disease other than tuberculosis (0=No; 1=Yes)? |__|

26. If yes, specify the disease: _____________________

27. Smoking (0=No; 1=Yes) ? |__| If yes, specify number of packs/year |__|

28. Cancer (0=No ; 1=Yes ) ? |If yes, specify the organ affected: _____________________

29. Other comorbidities, please specify:

____________________________________________________

30. Have you ever been affected by Covid-19 (0=No; 1=Yes)? |__|

31. If yes, which vaccine 1 (1=AstraZeneca; 2=Johnson and Johnson; 3=Sinopharm; 4=Pfizer,

5=Other)? |__| If other specify_____________________________________________

32. Number of doses |__|||__|

33. Date of Dose 1 Date :____ /___/____/202__ or |__||__||__|| weeks

34. Date of Dose 2 Date :____ /___/____/202__ or |__||__|||__| weeks

35. If yes, which vaccine 2 (1=AstraZeneca; 2=Johnson and Johnson; 3=Sinopharm; 4=Pfizer,

5=Other)? |__| If other specify_____________________________________________

36. Number of doses |__|||__|

37. Date of Dose 1 Date :____ /___/____/202__ or |__||__||__|| weeks

38. Date of Dose 2 Date :____ /___/____/202__ or |__||__|||__| weeks

**HIV INFORMATION:**

39. How long have you known your HIV status: Date:____ /___/____/202__

40. Are you on ARV treatment (0=No; 1=Yes)?

41. How long have you been on ARV treatment: Date:____ /___/____/202__

42. What is your initial treatment regimen (1=TLD(TDF/3TC/DTG); 2=TLE(TDF/3TC/EFV);

3=ABC/3TC/DTG; 4= ABC/3TC/EFV; 5=Other)? |__|

43. If other scheme, please specify_________________________________________

44. Have you ever changed your ARV treatment (0=No; 1=Yes)? |__|

45. If yes, what is your current treatment regimen (1=TLD(TDF/3TC/DTG);

2=TLE(TDF/3TC/EFV); 3=ABC/3TC/DTG; 4= ABC/3TC/EFV; 5=Other)? |__| If other

scheme, specify_________________________________________

46. How old is it? Date:____ /___/____/202__

47. Are you on Chemoprophylaxis (0=No; 1=Yes)?

48. If yes, which ones (1=Cotrimoxazole; 2=Isoniazide; 3=Other)? |__| If other chemoprophylaxis

to be specified __________________

49. Are you/have you been on treatment for any opportunistic infections (0=No; 1=Yes)?

If yes which one(s) :

50. Tuberculosis (0=No; 1=Yes) ?

51. Toxoplasmosis (0=No; 1=Yes) ?

52. Prurigo (0=No; 1=Yes) ?

53. Digestive candidiasis (0=No; 1=Yes) ?

54. Isosporosis (0=No; 1=Yes) ?

55. Cryptococcosis (0=No; 1=Yes) ?

56. Autre(s), préciser______________________________________________________________

57. When was your last viral load: Date:____ /___/____/202__

58. What was your last viral load result (0=Undetectable; 1=Detectable)? |__|

59. If detectable, specify the number : __________________ __________________

60. When was your last CD4 count: Date:____ /___/____/202__

61. What was your last CD4 count __________________

62. What was the participant's initial WHO stage (1=Stage 1; 2=Stage 2; 3=Stage 3; 4=Stage 4)?

|__|

63. What is the participant's current WHO stage (1=Stage 1; 2=Stage 2; 3=Stage 3; 4=Stage 4)? |__|

**IF HIV-TB CO-INFECTION**

64. How long have you known your TB status: Date:____ /___/____/202__

65. Specify TB type (1=TB S; 2=TB-MR; 3=TB-XR)? |__|

66. Specify the location of the TB (1= Pulmonary; 2=Extrapulmonary)

67. If extra pulmonary, specify __________________

68. Are you on anti-tuberculosis treatment (0=No; 1=Yes)? |__|

69. How long have you been on treatment : Date:____ /___/____/202__

70. What is your initial treatment regimen __________________

71. Have you ever changed your ARV treatment (0=No; 1=Yes)? |__|

72. If yes, what is your current treatment regimen __________________

73. How old is it? Date:____ /___/____/202__

74. When was your last bacilloscopy: Date:____ /___/____/202__

75. What was the result of your last bacilloscopy (0=Negative; 1=Positive)? |__|

76. When was your last Xpert MTB/RIF test Date:____ /___/____/202__

77. What was the result of this Xpert MTB/RIF test: (0=Negative; 1=Positive)?

**TREATMENT ADHERENCE**

78. Have you ever missed your follow-up appointments (0=Never; 1=Once/rarely; 2=Two

times/sometimes; 3=Three times/often; 4=More than three times/always)? |__|

79. If at least once, why? _______________________________________________________

80. Have you ever forgotten to take your medication (0=Never; 1=Once/rarely; 2=Two

times/sometimes; 3=Three times/often; 4=More than three times/always)? |__|

81. Is there someone who regularly reminds you to take your medication? (0=No; 1=Yes) |__|

**ACCESSIBILITY TO HEALTH SERVICES/OFFERS**

82. Has Covid-19 reduced your access to the various services/offerings necessary for your care?

(0=No; 1=Yes) |__|

83. With Covid-19 did you miss any follow-up appointments (0=No; 1=Yes)? |__|

84. With Covid-19 did you have any non-productive follow-up appointments (0=No; 1=Yes)? |__|

If so, why?

85. No staff (0=No; 1=Yes) ?

86. No consultation (0=No; 1=Yes) ?

87. Absence of paraclinical examination (0=No; 1=Yes) ?

88. Break in treatment (0=No; 1=Yes) ?

89. Autre, préciser _________________________________________________

**CLINICAL INFORMATION**

**Do you have these symptoms?**

90. Fever/Heat History (0=No; 1=Yes) ?

91. Headache (0=No; 1=Yes) ?

92. Cough since the beginning of the disease (0=No; 1=Yes) ?

93. Shortness of breath/dyspnea (0=No; 1=Yes)? |__|

94. Chest pain (0=No; 1=Yes) ?

95. Sore throat (0=No; 1=Yes) ?

96. Runny or stuffy nose (0=No; 1=Yes) ?

97. General Weakness/Asthenia (0=No; 1=Yes) ?

98. Nausea/vomiting (0=No; 1=Yes) ?

99. Diarrhea (0=No; 1=Yes) ?

100. Irritability / mental confusion (0=No; 1=Yes) ?

101. Loss of taste (0=No; 1=Yes) ?

**102.** Loss of sense of smell (0=No; 1=Yes) ?

103. Palpitation (0=No; 1=Yes) ?

**104.** Muscle pain (0=No; 1=Yes) ?

105. Abdominal pain (0=No; 1=Yes) ?

106. Joint pain (0=No; 1=Yes) ?

107. Other(s), specify ______________________________

108. If at least one symptom, specify onset of symptomatology ______ /___ /______

109. Have you consulted for these symptoms (0=No; 1=Yes)? |__|

110. If yes, what was the diagnosis ______________________________

111. If yes, what was the treatment

_______________________________________________________

112. Have you taken any traditional medicines for this symptomatology (0=No; 1=Yes)? |__|

113. If yes, which one(s)

_______________________________________________________

**114.** What are the participant's constants**:**

115. Temperature(°C) : |__||__| , |__|

116. Weight(kg) : |__||__|||

117. Size(cm) : |__||__|||

118. Respiratory rate (cycles/min): |__||__|

119. Sap02(%) : |__||__|

120. Pulse (beats/min): |__||__||

**POTENTIAL EXPOSURE TO COVID 19 IN THE LAST 14 DAYS (OR PRIOR TO SYMPTOM**

**ONSET)**

121. Have you traveled in the last 14 days (0=No; 1=Yes)? |__|

122. If yes, please specify where ........................

123. When? __ __/__ __/__ __; or approximate duration in days |__||

124. Have you been in contact with a confirmed or probable COVID-19 case in the past 14

days (0=No; 1=Yes)? |__|

125. If yes. Specify: The nature of the relationship with the contact (no

name).......................................

126. Place of contact? ......... ............ When? __ __/__ __/__ __ __ __; or approximate

duration in days |__||

127. Have you attended a mass gathering (e.g., wedding, baptism, market) in the past 14

days (0=No; 1=Yes)? |__|

128. If yes, please specify which one? ................................ Location

?...........................When? __ __/__ __/__ __

129. or the approximate duration in days |__|__|

130. Have you visited a health facility/traditional healer in the past 14 days (as a

patient/attendant)? (0=No; 1=Yes) |__|

131. If yes, please specify: Which institution? .................. Location ?...............................

132. When? __ __/__ __/__ __ __ __ ; or approximate duration in days |__||

133. Do you live in contact with animals? (0=No; 1=Yes) |__| if yes which

ones:.............................

134. Have you visited a live animal market in the past 14 days (0=No; 1=Yes)? |__|

135. Have you regularly worn a mask in public places in the past 14 days? (0=No; 1=Yes)

|__|

136. Have you regularly observed hand hygiene in the past 14 days (0=No; 1=Yes)? |__|

137. Have you regularly observed the distancing measures in public during the past 14 days

(0=No; 1=Yes)? |__|

**Evaluation of the impact of COVID-19 in people coinfected with HIV and/or tuberculosis in low-income countries: study protocol for mixed methods research in Burkina Faso: Patients with tuberculosis inclusion form**

**GENERAL INFORMATION**

1. Date of inclusion: __ __/__ __/2022

2. Name of the investigator .......................................

3. Participant ID: |__||__|||

**SOCIO-DEMOGRAPHIC CHARACTERISTICS**

4. Initial name(s): ___________________________

5. Date of birth: ____/____/______ or Age in years: ____

6. Gender: □ Female □ Male

7. Religion: **(**1=Muslim; 2=Christian; 3=Traditional; 4=Other)? |__| Other to specify __________

8. Occupation (1=Publicly Employed; 2=Privately Employed; 3=Farmer; 4=Farmer, 5=Trading,

6=Pupil/Student, 7=Housewife, 8=Informal Sector, 9=Retired, 10=Other)? |__| Other to specify

______________________________

9. District of Residence: ______________ City/Town: ______________ Area: __________

10. Place of residence: □ Urban / □ Rural

**UNDERLYING CONDITIONS AND CO-MORBIDITIES PRIOR TO STARTING ANTITUBERCULOSIS**

**TREATMENT,**

**IF**

**ANY**

1. Pregnancy (0=No; 1=Yes; 2=NA)? |__| If Yes, specify trimester: |__| (1,2,3 or 4)

2. Postpartum (0=No ; 1=Yes ; 2=NA) ? |__| Diabetes (0=No ; 1=Yes ) ? |__|

3. Liver disease (0=No ; 1=Yes ) ? |If yes, specify the disease: _____________________

4. Kidney disease (0=No ; 1=Yes ) ? |If yes, specify the disease: _____________________

5. Cardiovascular disease including known and treated hypertension (0=No ; 1=Yes )? |__|

6. If yes, specify the disease: _____________________

7. Chronic neurological or neuromuscular disease (0=No ; 1=Yes )? |__|

8. If yes, specify the disease: _____________________

9. Chronic lung disease other than tuberculosis (0=No; 1=Yes)? |__|

10. If yes, specify the disease: _____________________

11. Smoking (0=No; 1=Yes) ? |__| If yes, specify the number of packs/year |__|

12. Cancer (0=No ; 1=Yes ) ? |If yes, specify the organ affected: _____________________

13. HIV infection (0=No; 1=Yes; 2=DND)? |__|

14. If Yes How long have you known your HIV status: Date:____ /___/____/202__

15. Are you on ARV treatment (0=No; 1=Yes)?

16. How long have you been on ARV treatment: Date:____ /___/____/202__

17. What is your initial treatment regimen (1=TLD(TDF/3TC/DTG); 2=TLE(TDF/3TC/EFV);

3=ABC/3TC/DTG; 4= ABC/3TC/EFV; 5=Other)? |__|

18. If other, please specify_________________________________________

19. Have you ever changed your ARV treatment (0=No; 1=Yes)? |__|

20. If yes, what is your current treatment regimen (1=TLD(TDF/3TC/DTG);

2=TLE(TDF/3TC/EFV); 3=ABC/3TC/DTG; 4= ABC/3TC/EFV; 5=Other)? |__|

21. If other, please specify_________________________________________

22. How old is it? Date:____ /___/____/202__

23. Are you on Chemoprophylaxis (0=No; 1=Yes)?

24. If yes, which ones (1=Cotrimoxazole ; 2=Isoniazide ; 3=Other) If Other, please specify

__________________

25. Are you/have you been on treatment for any opportunistic infections (0=No; 1=Yes)?

26. If yes which one(s) :

27. Tuberculosis (0=No; 1=Yes) ?

28. Toxoplasmosis (0=No; 1=Yes) ?

29. Prurigo (0=No; 1=Yes) ?

30. Digestive candidiasis (0=No; 1=Yes) ?

31. Isosporosis (0=No; 1=Yes) ?

32. Cryptococcosis (0=No; 1=Yes) ?

33. Autre(s),

préciser_____________________________________________________________________

34. When was your last viral load: Date:____ /___/____/202__

35. What was your last viral load result (0=Undetectable; 1=Detectable)? |__|

36. If detectable, specify the number : __________________ __________________

37. When was your last CD4 count: Date:____ /___/____/202__

38. What was your last CD4 count __________________

39. What was the participant's initial WHO stage (1=Stage 1; 2=Stage 2; 3=Stage 3; 4=Stage 4)?

|__|

40. What is the participant's current WHO stage (1=Stage 1; 2=Stage 2; 3=Stage 3; 4=Stage 4)? |__|

41. Other comorbidities, please specify:

____________________________________________________

42. Have you ever been affected by Covid-19 (0=No; 1=Yes)? |__|

43. If yes, which vaccine 1 (1=AstraZeneca; 2=Johnson and Johnson; 3=Sinopharm; 4=Pfizer,

5=Other)? |__| If other specify_____________________________________________

44. Number of doses |__|||__|

45. Date of Dose 1 Date :____ /___/____/202__ or |__||__||__|| weeks

46. Date of Dose 2 Date :____ /___/____/202__ or |__||__|||__| weeks

47. If yes, which vaccine 2 (1=AstraZeneca; 2=Johnson and Johnson; 3=Sinopharm; 4=Pfizer,

5=Other)? |__| If other specify_____________________________________________

48. Number of doses |__|||__|

49. Date of Dose 1 Date :____ /___/____/202__ or |__||__||__|| weeks

50. Date of Dose 2 Date :____ /___/____/202__ or |__||__|||__| weeks

**INFORMATION ON TB:**

51. How long have you known your TB status: Date:____ /___/____/202__

52. Specify TB type (1=TB S; 2=TB-MR; 3=TB-XR)? |__|

53. Specify the location of the TB (1= Pulmonary; 2=Extrapulmonary)

54. If extra pulmonary, specify __________________

55. Are you on anti-tuberculosis treatment (0=No; 1=Yes)? |__|

56. How long have you been on treatment : Date:____ /___/____/202__

57. What is your initial treatment regimen __________________

58. Have you ever changed your TB treatment (0=No; 1=Yes)?

59. If yes, what is your current treatment regimen __________________

60. How old is it? Date:____ /___/____/202__

61. When was your last bacilloscopy: Date:____ /___/____/202__

62. What was the result of your last bacilloscopy (0=Negative; 1=Positive)? |__|

63. When was your last Xpert MTB/RIF test Date:____ /___/____/202__

64. What was the result of this Xpert MTB/RIF test: (0=Negative; 1=Positive)?

**TREATMENT ADHERENCE**

65. Have you ever missed your follow-up appointments (0=Never; 1=Once/rarely; 2=Two

times/sometimes; 3=Three times/often; 4=More than three times/always)? |__|

66. If at least once, why? _______________________________________________________

67. Have you ever forgotten to take your medication (0=Never; 1=Once/rarely; 2=Two

times/sometimes; 3=Three times/often; 4=More than three times/always)? |__|

68. Have you ever missed taking your medication (0=No; 1=Yes)? |__|

69. Have you ever forgotten to take your medication while traveling (0=No; 1=Yes)? |__|

70. Did you take your medication yesterday (0=Never; 1=Once/rarely; 2=Two times/sometimes;

3=Three times/often; 4=More than three times/always)? |__|

71. Have you ever stopped taking your medications because they put you under control (0=No;

1=Yes)? |__|

72. Do you find that taking the treatment every day is a problem that makes it difficult to stick to

the treatment plan? (0=No; 1=Yes) |__|

73. Do you have difficulty taking medications (0=Never; 1=Once/rarely; 2=Two

times/occasionally; 3=Three times/often; 4=More than three times/always)? |__|

74. Do you have trouble remembering when to take your medications? (0=Never; 1=Once/rarely;

2=Two times/sometimes; 3=Three times/often; 4=More than three times/always)? |__|

75. Have you ever stopped taking your medication (0=Never; 1=Once/rarely; 2=Two

times/sometimes; 3=Three times/often; 4=More than three times/always)? |__|

76. Is there someone who regularly reminds you to take your medication? (0=No; 1=Yes) |__|

77. Do you feel you can share your experience of the disease with other patients? (0=No; 1=Yes)

|__|

**ACCESSIBILITY TO HEALTH SERVICES/OFFERS**

78. Has Covid-19 reduced your access to the various services/offerings necessary for your care?

(0=No; 1=Yes) |__|

79. With Covid-19 did you miss any follow-up appointments (0=No; 1=Yes)? |__|

80. With Covid-19 did you have any non-productive follow-up appointments (0=No; 1=Yes)? |__|

If so, why?

81. Absence of staff (0=No; 1=Yes) ?

82. No consultation (0=No; 1=Yes) ?

83. Absence of paraclinical examination (0=No; 1=Yes) ?

84. Break in treatment (0=No; 1=Yes) ?

85. Autre, préciser _________________________________________________

**CLINICAL INFORMATION**

**Do you have these symptoms?**

86. Fever/Heat History (0=No; 1=Yes) ?

87. Headache (0=No; 1=Yes) ?

88. Cough since the beginning of the disease (0=No; 1=Yes) ?

89. Shortness of breath/dyspnea (0=No; 1=Yes)? |__|

90. Chest pain (0=No; 1=Yes) ?

91. Sore throat (0=No; 1=Yes) ?

92. Runny or stuffy nose (0=No; 1=Yes) ?

93. General Weakness/Asthenia (0=No; 1=Yes) ?

94. Nausea/vomiting (0=No; 1=Yes) ?

95. Diarrhea (0=No; 1=Yes) ?

96. Irritability / mental confusion (0=No; 1=Yes) ?

97. Loss of taste (0=No; 1=Yes) ?

98. Loss of sense of smell (0=No; 1=Yes) ?

99. Palpitation (0=No; 1=Yes) ?

100. Muscle pain (0=No; 1=Yes) ?

101. Abdominal pain (0=No; 1=Yes) ?

102. Joint pain (0=No; 1=Yes) ?

103. Other(s), specify ______________________________

104. If at least one symptom, specify onset of symptomatology ______ /___ /______

105. Have you consulted for these symptoms (0=No; 1=Yes)? |__|

106. If yes, what was the diagnosis ______________________________

107. If yes, what was the treatment

_______________________________________________________

108. Have you taken any traditional medicines for this symptomatology (0=No; 1=Yes)? |__|

109. If yes, which one(s)

_______________________________________________________

**110.** What are the participant's constants**:**

111. Temperature(°C) : |__||__| , |__|

112. Weight(kg) : |__||__|||

113. Size(cm) : |__||__|||

114. Respiratory rate (cycles/min): |__||__|

115. Sap02(%) : |__||__|

116. Pulse (beats/min): |__||__||

**TENTIAL EXPOSURE TO COVID 19 IN THE LAST 14 DAYS (OR PRIOR TO SYMPTOM**

**ONSET)**

**117.** Have you traveled in the last 14 days (0=No; 1=Yes)? |__|

118. If yes, please specify where ........................

**119.** When? __ __/__ __/__ __; or approximate duration in days |__||

**120.** Have you been in contact with a confirmed or probable COVID-19 case in the past 14

days (0=No; 1=Yes)? |__|

**121.** If yes. Specify: The nature of the relationship with the contact (no

name).......................................

**122.** Place of contact? ......... ............ When? __ __/__ __/__ __ __ __; or approximate

duration in days |__||

**123.** Have you attended a mass gathering (e.g., wedding, baptism, market) in the past 14

days (0=No; 1=Yes)? |__|

124. If yes, please specify which one? ................................Location?...........................When?

__ __/__ __/__ __

**125.** or the approximate duration in days |__|__|

**126.** Have you visited a health facility/traditional healer in the past 14 days (as a

patient/attendant)? (0=No; 1=Yes) |__|

**127.** If yes, please specify: Which institution?..................Location?...............................

128. When? __ __/__ __/__ __ __ __ ; or approximate duration in days |__||

129. Do you live in contact with animals? (0=No; 1=Yes) |__| if yes which

ones:.............................

**130.** Have you visited a live animal market in the past 14 days (0=No; 1=Yes)? |__|

**131.** Have you regularly worn a mask in public places in the past 14 days? (0=No; 1=Yes)

|__|

132. Have you regularly observed hand hygiene in the past 14 days (0=No; 1=Yes)? |__|

133. Have you regularly observed the distancing measures in public during the past 14 days

(0=No; 1=Yes)? |__|

**PARACLINICAL EXAMINATIONS**

134. Was a nasopharyngeal sample taken (0=No; 1=Yes)?

135. Was sputum with induced sputum collected (0=No; 1=Yes)?

136. Was venous blood 1 drawn (0=No; 1=Yes)? |__|

137. Was venous blood 2 drawn (0=No; 1=Yes)? |__|

138. Was a drop of dried blood collected on filter paper (0=No; 1=Yes)? |__|

139. Was a thick drop performed (0=No; 1=Yes)? |__|

140. Was a saliva sample taken (0=No; 1=Yes)?

141. Was a urine sample taken (0=No; 1=Yes)?

142. Was a stool sample taken (0=No; 1=Yes)?
